# The Gender Gap in Aortic Dissection: A Prospective Analysis of Risk and Outcomes

**DOI:** 10.2478/jccm-2023-0024

**Published:** 2023-07-31

**Authors:** Cosmin Banceu, Marius Harpa, Klara Brinzaniuc, Ioan Tilea, Andreea Varga, Mirela Liana Gliga, Septimiu Voidazan, Nicolae Neagu, Dan Alexandru Szabo, Diana Banceu, Daiana Cristutiu, Ionut Alexandru Balmos, Alexandra Puscas, Marvin Oprean, Horatiu Suciu

**Affiliations:** George Emil Palade University of Medicine, Pharmacy, Science, and Technology of Targu Mures, Romania; I.O.S.U.D George Emil Palade University of Medicine, Pharmacy, Science, and Technology of Targu Mures, Romania; Emergency Institute for Cardiovascular Diseases and Transplantation Targu Mures, Romania; Dimitrie Cantemir University of Targu Mures, Romania

**Keywords:** Stanford type A acute aortic dissection, aortic dissection, gender gap, risk factors, in-hospital mortality

## Abstract

Aortic dissection (AD) is a severe cardiovascular condition that could have negative consequences. Our study employed a prospective design and examined preoperative, perioperative, and postoperative data to evaluate the effects of gender on various medical conditions. We looked at how gender affected the results of aortic dissection (AD). In contrast to female patients who had more systemic hypertension (p=0.031), male patients had higher rates of hemopericardium (p=0.003), pulmonary hypertension (p=0.039), and hemopericardium (p=0.003). Dobutamine administration during surgery significantly raised the mortality risk (p=0.015). There were noticeably more women patients (p=0.01) in the 71 to 80 age group. Significant differences in age (p=0.004), eGFR at admission (p=0.009), and eGFR at discharge (p=0.006) were seen, however, there was no association between gender and mortality. In conclusion, our findings highlight that gender may no longer be such an important aspect of aortic dissection disease as we previously thought, and this information could have an important contribution for surgeons as well as for anesthesiologists involved in the management of acute aortic dissection.

## Introduction

Aortic dissection, a life-threatening cardiovascular condition remains an important issue for patients and healthcare systems worldwide [1]. Since risk factors might prolong patient time spent in the intensive care unit (ICU), identifying them at any point of hospitalization could dictate medical evolution and decrease the mortality rate [2].

Research has shown higher in-hospital mortality rates for cardiac procedures compared with general surgeries, and women are more likely to die following cardiac procedures than men [3,4,5]. These data averages suggest that it is crucial to take into consideration patient heterogeneity and other factors that could impact overall clinical outcomes [6,7].

The overall likelihood of outcome in cardiac surgery, including surgery for various heart conditions, can often range from 2% to 7% and might be influenced by factors such as race, gender, age, low cardiac output, and comorbidities not related to heart disease [8]. However, mortality rate might increase above 60% when patients develop postoperative acute kidney injury (AKI) [9,10]. Even though cardiac surgery originated in 1886, currently, more than 6.4 billion people do not have easy access to treatment, and it is imperative to understand the factors correlated with high mortality risk [11,12,13].

Cardiac surgery has unique characteristics, such as high rates and volumes of exogenous blood product transfusion, and systemic inflammatory response (SIR) after cardiopulmonary bypass (CPB), that make patients more susceptible to AKI [14]. CPB in cardiac surgery efficiently sustains myocardial oxygenation and systemic blood oxygen levels by diverting blood flow away from the heart while using an artificial lung (oxygenator) for effective gas exchange [15,16,17]. Improper stimulation of inflammatory markers generated by extracorporeal circulation following cardiac surgery continues to be a common cause of postoperative complications, including AKI [15]. This aspect could lead to a glomerular filtration rate (eGFR) < 30–44 mL/min/1.73 m^2^, which is associated with increased inhospital and long-term mortality rates [18,19,20,21].

Studies have shown a strong correlation between mortality rate and a drop in eGFR, where negative outcome increase to 14% for each 10 mL/min/1.73 m^2^ decline in eGFR [22,23,24].

Countless factors, including patient characteristics, type of procedure, and perioperative management, could influence variations in serum creatinine (SCr) levels after cardiac surgery [25]. Some preliminary data indicate there are gender variations in the prevalence of AKI and changes in SCr levels following AD surgery treatment [7,26,27].

Starting from admission, SCr levels could be a predictor marker for aortic surgery mortality rates, considering the fact that endogenous filtration of this biomarker was used to determine eGFR, also including urinary output [28].

Elevated creatinine levels have been tied directly to higher mortality, particularly in women with AKI, several research findings highlight the significance of accepting gender differences in the management of this medical condition [29,30].

According to different studies, female mortality rates following hemofiltration for AKI were higher than in males [31]. Although causes of gender differences are not fully understood, they could include differences in comorbidities, severity of the disease, or hormonal factors [32,33].

The cardiovascular system and the renal system are closely linked, and preoperative kidney malperfusion, atherosclerosis, systemic inflammation, hemopericardium, tamponade cardiac, and cardiogenic shock are all significantly related to the progression of AKI [17, 34]. This exemplifies how important it is to fully understand how cardiac and renal systems interplay immediately and how AKI progresses, considering that it is an extremely risky complication with an incidence rate of up to 18–67%, following cardiac surgery [31, 34,35].

Increased morbidity, mortality, and prolonged hospitalization in ICU are associated with AKI [36]. Recent studies have demonstrated significant differences between genders and the impact of AKI following cardiac surgery, where female patients present increased susceptibility for developing such nephropathy versus male patients [6, 31]. Gender-specific research on AKI following cardiac surgery is continuing to expand, but the approaches behind each of the inequalities are still incompletely known and require additional studies [31,35]. Researchers need to develop efficient prevention and therapeutic strategies to fully understand the evolution of AD, risk factors, and clinical outcomes related to the gender gap in AKI following surgical treatment of this specific medical condition [1,2,3,4,5].

Women over 75 are more at risk for death following cardiac surgery than men [26,37]. Different comorbidities and postoperative complications are only a few of the possible reasons for gender differences [32]. The gender gap must be recognized, and efficient strategies developed to improve outcomes, especially for older female patients [31,33].

Inotropic and vasoconstrictor drugs, typically utilized preoperatively for persistent hypotension and postoperatively to manage vasoplegia associated with a systemic inflammatory syndrome, are related to renal dysfunction in aortic dissection, particularly in female patients [38].

Consequences of mechanical ventilation on hemodynamic and renal perfusion during surgical treatment of aortic dissection could lead to AKI [2]. Female patients with aortic dissection may be more vulnerable due to lower baseline cardiac output and aortic diameters, necessitating close monitoring of renal function during mechanical ventilation [6,39,33].

Due to comorbidities, severity of the disease or different treatment responses, female patients with aortic dissection may require to stay longer time in ICU versus male patients [4,27]. To maximize results in AD treatment, doctors should consider the gender gap in each management strategy and establish individualized care approaches [6]. To better understand the underlying factors for gender differences in mortality rates of AD and develop specific approaches that would improve patient outcomes, further studies are required.

Aortic diseases continue to have deadly outcomes despite significant technological advances in therapy [33,40,41]. The main aim of this research was to analyse in a prospective study how the gender gap could impact mortality rates among patients with aortic dissection.

## Materials and Methods

We had three hypotheses in our study:

1. There is a statistically significant difference between patients with Stanford A dissection associating acute kidney injury and the gender gap.
2. There are statistically significant differences between utilization of inotropic drugs, bypass, ischemia time, time spent in ICU or intubation time and patients with Stanford A dissection considering the gender gap.
3. There are statistically significant differences between gender and in-hospital and immediate mortality.

We included in this prospective study 101 patients with Stanford A thoracic dissection treatment between 2019 and 2022. The included criteria were SCr levels and eGFR for every patient at three different time points: at admission, on the first day in the ICU, and at discharge, also inotropic drugs used preoperatively, perioperatively, and postoperatively, including cross-clamp and CPB time, intubation period, and days spent in ICU, all analysed for the two groups, men versus women.

Patients whose consent could not be obtained or who refused surgery were excluded from the study. We also excluded patients with chronic cardiac failure with functional status, chronic renal failure necessitating replacement therapy, tubulointerstitial diseases (urinary-tract infections, toxic effects of drugs, nephrolithiasis, urinary-tract obstruction) or history of renal transplant patients with neoplasia, systemic infections, autoimmune disease, patients with nephrotoxic antibiotics, and those under 18 years old. Data were analysed in agreement with the ethical code and current legal requirements. All manoeuvres were performed using local protocols during surgery and in ICU.

In-hospital mortality is the term we use to describe patients who died while hospitalized and immediate mortality regards the patient in the first six months after discharge.

The study was conducted following the Declaration of Helsinki and approved by ethical university resolution No. 7225/07.10.2019, No. 878/23.04.2020, and 1359/10.05.2021.

Regarding statistical analysis, continuous variables were expressed by descriptive statistics as mean ± standard deviation (SD) or median and minimum-maximum, while the categorical variables were summarized by absolute and relative frequencies. All continuous variables were checked for normality using the Kolmogorov–Smirnoff test.

The variables that have a Gaussian distribution (ex. age), were interpreted by means and SD, and the student's *t*-test was applied. Variables without a Gaussian distribution, i.e., days in the ICU were analyzed by median and minimum-maximum, and the Mann–Whitney test was applied.

For all two-sided statistical tests, the significance was achieved if the estimated significance level *p*-value was ≤ 0.05. Statistical analysis was performed using MedCalc Software, Version 12.5.0.0.

## Results

The study reflects the characteristics and outcomes of cardiac patients following acute aortic dissection surgical treatment. In-hospital mortality, immediate mortality, renal disease, hemofiltration, hemopericardium, cardiac tamponade, cardiogenic shock, hypertension, myocardial infarction, cardiac arrest, pulmonary hypertension, chronic obstructive pulmonary disease, asthma, and use of inotropic therapy are the factors presented (preoperatively, perioperatively, and postoperatively) in Table 1.

**Table 1.** Detailed clinical and baseline characteristics of research participants

**Variables**	**Total (101)**	**Female (35)**	**Male (66)**	**OR(CI95%)**	**p-value**
In-hospital mortality	41 (40.6)	16 (45.7)	25 (37.9)	0.72 (0.31–1.66)	0.290^*^
Immediate mortality	11 (10.9)	6 (9.1)	5 (14.3)	0.6 (0.16–2.12)	0.315^*^
Renal dysfunction	53 (52.5)	37 (56.1)	16 (45.7)	1.51 (0.66–3.45)	0.217^*^
Hemofiltration	15 (14.9)	9 (13.6)	6 (17.1)	0.76 (0.24–2.35)	0.770^*^
Hemopericardium	35 (34.7)	16 (24.2)	19 (54.3)	0.26 (0.11–0.64)	0.003^*^
Cardiac tamponade	14 (13.9)	9 (13.6)	5 (14.3)	0.94 (0.29–3.08)	0.575^*^
Cardiogenic shock	11 (10.9)	6 (9.1)	5 (14.3)	0.60 (0.16–2.12)	0.315^*^
Systemic hypertension	74(73.3)	44 (66.7)	30 (85.7)	0.33 (0.11–0.97)	0.031^*^
Myocardial infarction	2 (2)	1 (1.5)	1 (2.9)	0.52 (0.32–8.62)	0.575^*^
Cardiac arrest	2 (2)	1 (1.5)	1 (2.9)	0.52 (0.32–8.62)	0.575^*^
Pulmonary hypertension	3 (3)	0 (0)	3 (8.6)	0.32 (0.24–0.43)	0.039^*^
Chronic obstructive pulmonary disease	8 (7.9)	6 (9.1)	2 (5.7)	1.65 (0.31–8.64)	0.431^*^
Asthma	3 (3)	2 (3)	1 (2.9)	1.06 (0.09–12.1)	0.725^*^
pre-op Inotropes therapy	12 (11.9)	9 (13.6)	2 (8.6)	1.16 (0.42–6.67)	0.536^*^
Dopamine	3 (3)	2 (3)	1 (1.29)	1.06 (0.09–12.14)	0.725^*^
Dobutamine	7 (6.9)	5 (7.6)	2 (5.7)	1.35 (0.24–7.35)	0.539^*^
Adrenaline	2 (2)	1 (1.5)	1 (2.9)	0.52 (0.32–8.62)	0.575^*^
Noradrenaline	7 (6.9)	7 (10.6)	0 (0)	0.62 (0.53–0.73)	0.045^*^
peri-op Inotropes therapy	40 (39.6)	24 (36.4)	16 (45.7)	1.47 (0.64–2.29)	0.398^*^
Dopamine	63 (62.4)	44 (66.7)	19 (54.3)	0.59 (0.25–1.37)	0.281^*^
Dobutamine	79 (78.2)	47 (71.2)	32 (91.4)	4.31 (1.17–15.78)	0.015^*^
Adrenaline	86 (85.1)	54 (81.8)	32 (91.4)	2.37 (0.62–9.04)	0.159^*^
Noradrenaline	53 (52.5)	34 (51.5)	19 (54.3)	1.11 (0.49–2.54)	0.478^*^
post-op Inotropes therapy	47 (46.5)	30 (45.5)	17 (48.6)	1.13 (0.49–2.57)	0.464^*^
Dopamine	66 (65.3)	47 (71.2)	19 (54.3)	0.48 (0.20–1.12)	0.070^*^
Dobutamine	75 (74.3)	47 (71.2)	28 (80.0)	1.61 (0.60–4.32)	0.237^*^
Adrenaline	82 (81.2)	54 (81.8)	28 (80.0)	0.88 (0.31–2.51)	0.511^*^
Noradrenaline	61 (60.4)	39 (59.1)	22 (62.9)	1.17 (0.50–2.72)	0.440^*^

* chi-square test; pre-, peri-post-op- preoperatively, perioperatively, and postoperatively

The analyses were presented for the overall group as well as separately by gender. The p-value indicates the statistical significance of differences observed in each variable between male and female patients. The strength and direction of the association between each variable and the outcome are provided as an OR (odds ratio) with a 95% CI (confidence interval). Overall, the data indicate that there were no significant differences between male and female patients in terms of in-hospital and immediate mortality. So far, while female patients were more likely to have hypertension, male patients had a greater probability to develop hemopericardium and pulmonary hypertension. Dobutamine treatment during post-operative surgical intervention was attributed to considerably increased mortality risks. A significantly lower mortality risk was attributed to the use of noradrenaline during peri-op and post-op surgical treatment. The use of different types of inotropic therapy or the gender of patients was not identified as being significantly different in other variables.

The patient distribution by age and gender in addition to corresponding p-values is presented in Table 2.

**Table 2.** Age characteristics of research participants

**Age**	**Total n=/%**	**Female n=/%**	**Male n=/%**	**p-value**
<50	12 (11.9%)	2 (5.7%)	10 (15.2%)	0.28
51–60	17 (16.8%)	5 (14.3%)	12 (18.2%)	0.82
61–70	43 (42.6%)	12 (34.3%)	31 (47.0%)	0.30
71–80	29 (28.7%)	16 (45.7%)	13 (19.7%)	0.01

There were 101 patients in total, grouped into four age groups: under 50, between 51 and 60, between 61 and 70, and between 71 and 80. For each age group, gender distribution was also highlighted. There was no significant difference in the gender distribution between men and women in the age groups under 50 years and 51 to 60 years. Gender distribution was statistically significant for the 71–80 age group, which included significantly more females than males. These results indicate that patient gender distribution in this study may be influenced by age, with more female patients than male patients in the 71–80 range of age.

Descriptive statistics were performed, including mean and standard deviation, for the overall group as well as separately by gender, see Table 3.

**Table 3.** Laboratory data of patients

**Variables**	**Total (n=101)**	**Female (n=35)**	**Male (n=66)**	**p-value**
Age	64.30 (+/−10.4)	68.31 (+/9.49)	62.17 (+/10.39)	0.004^*^
Ejection fraction	52 (+/−6.5)	51 (+/−6.65)	52 (+/−6.47)	0.58^*^
Creatinine at admission	1.16 (+/−0.44)	1.08 (+/−0.39)	1.21 (+/−0.45)	0.17^*^
eGFR at admission	71.30 (+/−23.4)	63.20 (+/−21.2)	75.59 (+/−23.53)	0.009^*^
Creatinine 1 day in the ICU	1.45 (+/−0.99)	1.08 (+/−0.39)	1.40 (+/−0.9)	0.994^*^
eGFR in the ICU	64.7 (+/−28.62)	54.63 (+/−26.57)	70.07 (+/−25.5)	0.024^*^
Creatinine at discharge	1.31 (+/−1.05)	1.47 (+/−1.48)	1.23 (+/−0.74)	0.638^*^
eGFR at discharge	72.29 (+/−28.25)	61.80 (+/−27.34)	77.85 (+/−27.31)	0.006^*^
Ischemia time	135.65 (+/−48.31)	131.17 (+/−47.1)	138.03 (+/−49.1)	0.50^*^
Total bypass time	201.73 (+/−73.35)	188.77 (+/−67.9)	208.61 (+/−75.6)	0.19^*^
Days in the ICU	1 (0–34)	2 (0–21)	1 (0–34)	0.88^**^
Intubation time	10 (1–792)	2 (0–21)	11 (1–792)	0.19^**^

* Student's *t*-test expressed data by mean+/−SD:

** Mann Whitney test, data expressed by median (min-max);

Age, ejection fraction, SCr levels, and eGFR at admission and discharge are the characteristics of patients. The results indicate that there were no significant differences in ejection fraction, creatinine at admission, SCr level day one in the ICU, SCr level at discharge, or eGFR at discharge, but that there were statistically significant differences between males and females in terms of age (p=0.004) and eGFR at admission (p=0.009).

These findings indicate that age and eGFR at admission should be considered with caution when analysing patient outcomes and deciding treatments, paying attention to gender differences, and these results are lending significant scientific credibility to hypothesis 1.

The mean ischemia time and total bypass time, as well as their associated standard deviations for every group, are part of the data provided in this research. The results demonstrated that the average ischemia time between the groups was not significantly different. The mean total CPB time among these groups was not significantly different. These results suggest that there may not be a substantial difference among the groups in the cross-clamp and CPB time.

The statistical methods were applied to analyse any possible variations in the field of anaesthesia support in light of the gender gap; see Table 4.

**Table 4.** Statistical analysis of the patient's risk factors according to their gender

**Variables**	**p-value**	**OR(CI95%)**
Gender	0.23	0.348 to 1.294
Intubation time	0.09	0.989 to 1.000
Ischemia time	0.93	0.986 to 1.012
CPB time	0.43	0.994 to 1.012

A Cox regression model with a p-value that was close to the statistical significance (p=0.098) indicated that the time of intubation had no significant influence on in-hospital mortality based on gender.

Noradrenaline significantly increased the required time for cardiac surgery (p=0.018), had a negative impact on in-hospital mortality (p=0.37), and was influenced by the gender of the patient, according to Cox regression analysis.

At this point, we could say that these results lend significant scientific credibility to hypothesis 2.

**Fig. 1.**
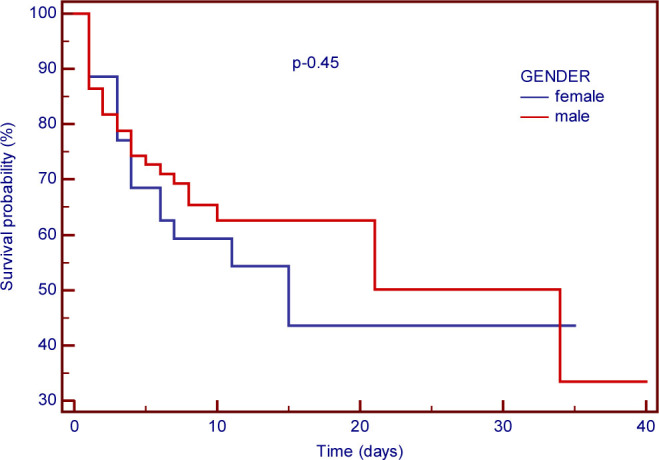
In-hospital mortality survival rate based on gender interpretation.

Figure 1 provides a graphic representation of Kaplan-Meier survival analysis and was utilised to evaluate in-hospital mortality.

The survival rate at day 1 was 88.6%+/−5.3% for females and 81.8%+/−4.7% for males, differentiated by gender. Survival rate was 59.3%+/−8.4% for males and 59.0%+/−8.4% for females at day 7 post-operation, with no statistically significant difference between the two groups being observed (p=0.45, OR 0.79 [0.4–1.5]).

There were 35 patients in the female group, and 6 of them died, giving the death event proportion of 9.1% for this group of patients regarding immediate mortality. A total of 5 deaths occurred among the 66 patients in the male group, for a mortality event percentage of 14.3%, with no statistically significant differences in the immediate mortality. No Kaplan-Meier survival analysis was performed due to the small sample size. Based on these findings, we can conclude that the third hypothesis is unsubstantiated.

## Discussion

Aortic dissection is a deadly cardiovascular disease that has an immediate impact on a patient's life. It is important to understand every factor that directly affects mortality rates [40,41].

Aortic dissection management has been studied in the context of gender, and there is evidence that men have a much higher risk than women [33,44,^42^,^43^]. Such studies helped to develop preventative and curative strategies for this life-threatening illness [4,5,32,33, 36].

It is essential to identify additional risk factors that may contribute to these disparities beyond the severity of the condition and its impact on treatment outcomes in a more complete understanding of gender differences regarding mortality rates related to aortic dissection management. Intubation, ICU, cross-clamp, and CPB time, and acute kidney injury are just a list of the potential factors that have been examined. These factors may have different degrees of influence on mortality rates if we take into consideration the gender of patients [5, 31, 36].

According to several studies, men might have an increased risk of developing undesirable outcomes following aortic surgery, such as high mortality rates and relatively long hospitalizations [42,43]. Also, following aortic surgery treatment, men might develop AKI, need more inotropic drugs, or longer periods of CPB [33, 44].

Our findings suggest that males had a greater tendency to develop hemopericardium and pulmonary hypertension, while females were more prone to high systemic hypertension. Regarding inotropic drugs used postoperatively, dobutamine was significantly associated with increased mortality risks. Meanwhile, administration perioperatively or postoperatively, noradrenaline was significantly associated with decreased mortality risks. Notably, neither the usage of different inotropic or vasoactive drug therapies nor the impact of gender on any other variables indicated significant differences. Also, there were no significant differences in in-hospital and immediate mortality between male and female patients.

Due to comorbidities, age plays a significant influence on anticipated mortality in aortic dissection [5,26]. Age should therefore be considered when evaluating the severity and expected outcomes of aortic disease in medical practice [26, 37].

According to our analysis, there were no significant differences in the gender distribution of patients between the age category of 51 to 60 and under 50. For the 71–80 age range, however, the gender distribution was statistically significant, with significantly more females than males. These results suggest that age could have a significant repercussion on the gender distribution of study participants, with a higher proportion of female than male group members in the 71–80 age category.

The patients’ gender varied significantly, according to the analysis of the population's health status and surgical treatment, certain patients needing more intensive care support and longer recovery time than others. Genetics, lifestyle, and overall health are only a few of the causes of this gender gap. For this reason, it is important to consider gender as a variable when planning treatments and preventive strategies that encourage equitable health outcomes [33, 40,41,43].

Our analysis reveals statistically significant differences regarding age and eGFR at admission between males and females, but no positive correlation based on gender difference regarding ejection fraction, CPB and ischemia time, SCr levels at admission, after one day in the ICU, at discharge, or eGFR at discharge. Management of patients with aortic dissection should take into consideration age and eGFR at admission as risk factors.

Aortic dissection has alarmingly high mortality rates especially if patients would fail to reach the hospital in time for prompt and effective treatment, so constant attention and concern for this disease are required [33, 40,41,43]. It is important to take into consideration preoperative and postoperative risk factors, and the gender gap to improve aortic dissection management and decrease their impact on the outcome [7, 25]. A focus on eligible risk factors is critical in emergency cases and their identification and good management might influence the mortality rates [38].

Regarding patients following aortic dissection surgery treatment, in-hospital mortality is a significant consequence of concern, so it is crucial to establish contributing factors. According to our study, there was no statistically significant difference in the mortality rates of male and female patients, highlighting that gender may not be a significant factor in predicting inhospital mortality rates.

The data analysis on immediate mortality shows that there were no statistically significant differences between the gender groups and mortality recorded in the follow-up period,

This study provides valuable insights into the characteristics and outcomes of patients undergoing aortic dissection surgery, highlighting the importance of considering various variables such as gender, age, inotropic therapy use, and renal function in predicting patient outcomes. These findings could assist clinical decisions and lead to better patient outcomes. Our research contributes to the wealth of knowledge already available on the subject and might have implications for further research in this field. If so far it has been considered that gender male has an important contribution to the incidence and mortality rate of aortic dissection, this it seems to be changing, and the gender gap is not as evident as it used to be.

### Limitations

It is crucial to remember that the study was limited by the exclusion of patients who died suddenly before being admitted, which might have influenced its outcomes. The lack of national registers of people who had surgeries renders it exceedingly hard to keep a record of these patients, consequently, follow-up is only allowed six months after surgery.

## Conclusions

In conclusion, there has been an upgrade regarding the way gender gaps in aortic dissection incidence and mortality have been viewed. The gender gap is not as large as previously believed according to this study, which also found that there were no appreciable differences in in-hospital and immediate mortality between male and female patients. However, more research is required to completely understand how gender affects aortic dissection outcomes taking the gender gap into account. The findings of this study, which consider factors like gender, age, usage of inotropic treatment, renal function, and mortality rate, can guide clinical judgement and enhance acute aortic dissection patient outcomes. The results presented might have a beneficial effect on following cardiac surgery studies and also on the decision about the most efficient medical therapies for this life-threatening disease known as acute aortic dissection.

## References

[1] Juraszek A, Czerny M, Rylski B (2022). Update in aortic dissection. Trends Cardiovasc Med.

[2] Liu K, Hao GW, Zheng JL (2020). Effect of Sequential Noninvasive Ventilation on Early Extubation After Acute Type A Aortic Dissection. Respir Care.

[3] Du W, Glasgow N, Smith P (2018). Major inpatient surgeries and in-hospital mortality in New South Wales public hospitals in Australia: A state-wide retrospective cohort study. Int J Surg.

[4] Nienaber CA, Fattori R, Mehta RH (2004). International Registry of Acute Aortic Dissection. Gender-related differences in acute aortic dissection. Circulation.

[5] Huckaby LV, Sultan I, Trimarchi S (2022). Sex-Based Aortic Dissection Outcomes From the International Registry of Acute Aortic Dissection. Ann Thorac Surg.

[6] Gasser S, Stastny L, Kofler M (2022). Type A aortic dissection is more aggressive in women. Eur J Cardiothorac Surg.

[7] Kennedy JW, Kaiser GC, Fisher LD (1981). Clinical and angiographic predictors of operative mortality from the collaborative study in coronary artery surgery (CASS). Circulation.

[8] Chertow GM, Lazarus JM, Christiansen CL (1997). Preoperative renal risk stratification. Circulation.

[9] Chertow GM, Levy EM, Hammermeister KE, Grover F, Daley J (1998). Independent association between acute renal failure and mortality following cardiac surgery. Am J Med.

[10] Zilla P, Yacoub M, Zühlke L (2018). Global Unmet Needs in Cardiac Surgery. Glob Heart.

[11] Ronco C, Bellomo R, Kellum JA (2019). Acute kidney injury. Lancet.

[12] Husain-Syed F, Ferrari F, Sharma A (2019). Persistent decrease of renal functional reserve in patients after cardiac surgery-associated acute kidney injury despite clinical recovery. Nephrol Dial Transplant.

[13] O’Neal JB, Shaw AD, Billings FT (2016). Acute kidney injury following cardiac surgery: current understanding and future directions. Crit Care.

[14] Ortega-Loubon C, Fernández-Molina M, Carrascal-Hinojal Y, Fulquet-Carreras E (2016). Cardiac surgery-associated acute kidney injury. Ann Card Anaesth.

[15] Meersch M, Schmidt C, Hoffmeier A (2017). Erratum to: Prevention of cardiac surgery-associated AKI by implementing the KDIGO guidelines in high risk patients identified by biomarkers: the PrevAKI randomized controlled trial. Intensive Care Med.

[16] Wu HB, Ma WG, Zhao HL (2017). Risk factors for continuous renal replacement therapy after surgical repair of type A aortic dissection. J Thorac Dis.

[17] Hansen MK, Gammelager H, Jacobsen CJ (2015). Acute Kidney Injury and Long-term Risk of Cardiovascular Events After Cardiac Surgery: A Population-Based Cohort Study. J Cardiothorac Vasc Anesth..

[18] Inker LA, Astor BC, Fox CH (2014). KDOQI US commentary on the 2012 KDIGO clinical practice guideline for the evaluation and management of CKD. Am J Kidney Dis.

[19] Holzmann MJ, Ahnve S, Hammar N (2005). Creatinine clearance and risk of early mortality in patients undergoing coronary artery bypass grafting. J Thorac Cardiovasc Surg.

[20] Saratzis A, Joshi S, Benson RA (2020). Acute Kidney Injury (AKI) in Aortic Intervention: Findings From the Midlands Aortic Renal Injury (MARI) Cohort Study. Eur J Vasc Endovasc Surg.

[21] Wang Y, Bellomo R (2017). Cardiac surgery-associated acute kidney injury: risk factors, pathophysiology and treatment. Nat Rev Nephrol.

[22] Ko T, Higashitani M, Sato A (2015). Impact of Acute Kidney Injury on Early to Long-Term Outcomes in Patients Who Underwent Surgery for Type A Acute Aortic Dissection. Am J Cardiol.

[23] Kao KD, Lee SKC, Liu CY, Chou NK (2022). Risk factors associated with longer stays in cardiovascular surgical intensive care unit after CABG. J Formos Med Assoc.

[24] Maheshwari K, Turan A, Mao G (2018). The association of hypotension during non-cardiac surgery, before and after skin incision, with postoperative acute kidney injury: a retrospective cohort analysis. Anaesthesia.

[25] Norton EL, Kim KM, Fukuhara S (2021). Differences among sexes in presentation and outcomes in acute type A aortic dissection repair. J Thorac Cardiovasc Surg.

[26] Chew STH, Hwang NC (2019). Acute Kidney Injury After Cardiac Surgery: A Narrative Review of the Literature. J Cardiothorac Vasc Anesth.

[27] Khwaja A (2012). KDIGO clinical practice guidelines for acute kidney injury. Nephron Clin Pract..

[28] Parikh CR, Puthumana J, Shlipak MG (2017). Relationship of Kidney Injury Biomarkers with Long-Term Cardiovascular Outcomes after Cardiac Surgery. J Am Soc Nephrol.

[29] Conlon PJ, Stafford-Smith M, White WD (1999). Acute renal failure following cardiac surgery. Nephrol Dial Transplant.

[30] Divchev D, Najjar T, Tillwich F (2015). Predicting long-term outcomes of acute aortic dissection: a focus on gender. Expert Rev Cardiovasc Ther.

[31] Schiffl H (2020). Gender differences in the susceptibility of hospital-acquired acute kidney injury: more questions than answers. Int Urol Nephrol.

[32] Carbone A, Ranieri B, Castaldo R (2023). Sex Differences in Type A Acute Aortic Dissection: A Systematic Review and Meta-Analysis. Eur J Prev Cardiol.

[33] Nishigawa K, Fukui T, Uemura K, Takanashi S, Shimokawa T (2020). Preoperative renal malperfusion is an independent predictor for acute kidney injury and operative death but not associated with late mortality after surgery for acute type A aortic dissection. Eur J Cardiothorac Surg.

[34] Wang L, Zhong G, Lv X (2022). Risk factors for acute kidney injury after Stanford type A aortic dissection repair surgery: a systematic review and meta-analysis. Ren Fail.

[35] Brar A, Markell M (2019). Impact of gender and gender disparities in patients with kidney disease. Curr Opin Nephrol Hypertens.

[36] Morjan M, Mestres CA, Lavanchy I (2022). The impact of age and sex on in-hospital outcomes in acute type A aortic dissection surgery. J Thorac Dis.

[37] Zhang K, Shang J, Chen Y (2021). The prognosis and risk factors for acute kidney injury in high-risk patients after surgery for type A aortic dissection in the ICU. J Thorac Dis.

[38] Zhou Y, Peng W, Yang G (2021). Gender Difference is Associated with Short-Term Outcomes in Non-Surgically Managed Acute Aortic Dissection Patients with Hypertension: A Retrospective Cohort Study. Risk Manag Healthc Policy.

[39] Rodríguez-Lecoq R, Maeso J, Bellmunt S (2021). Changes in the diagnosis and management of acute aortic syndrome and associated mortality in the last 20 years. Rev Esp Cardiol (Engl Ed).

[40] Czerny M, Schoenhoff F, Etz C (2015). The Impact of Pre-Operative Malperfusion on Outcome in Acute Type A Aortic Dissection: Results From the GERAADA Registry. J Am Coll Cardiol.

[41] Wang X, Ren HM, Hu CY (2016). Predictors and in-hospital outcomes of preoperative acute kidney injury in patients with type A acute aortic dissection. J Geriatr Cardiol.

[42] Suzuki T, Asai T, Kinoshita T (2018). Clinical differences between men and women undergoing surgery for acute Type A aortic dissection. Interact Cardiovasc Thorac Surg.

[43] Chang CY, Wu CF, Muo CH (2023). Sex Differences in Temporal Trends and Risk Factors of Aortic Dissection in Taiwan. J Am Heart Assoc.

